# Physicochemical and Functional Properties of Skipjack Tuna (*Katsuwonus pelamis*) Bone Gelatin Extracted at Different Temperatures

**DOI:** 10.3390/foods14132256

**Published:** 2025-06-26

**Authors:** Zhixin Rao, Haohao Shi, Jiamei Wang, Guanghua Xia

**Affiliations:** 1 Hainan Engineering Research Center of Aquatic Resources Efficient Utilization in South China Sea, Key Laboratory of Food Nutrition and Functional Food of Hainan Province, Key Laboratory of Seafood Processing of Haikou, College of Food Science and Technology, Hainan University, Haikou 570228, China; 20223007588@hainanu.edu.cn (Z.R.); shihaohao@hainanu.edu.cn (H.S.); 992918@hainanu.edu.cn (J.W.); 2 Collaborative Innovation Center of Provincial and Ministerial Co-Construction for Marine Food Deep Processing, Dalian Polytechnic University, Dalian 116034, China

**Keywords:** Skipjack tuna bone gelatin, extraction temperature, gel strength, rheological properties, antioxidant activity

## Abstract

In this study, we produced gelatin from Skipjack tuna (*Katsuwonus pelamis*) bone. We used three heating levels to obtain the gelatin (80 °C, 90 °C, 100 °C), and its physicochemical and gel characteristics were thoroughly examined. The results revealed that the extraction temperature significantly affected the yield, gel strength, amino acid composition, antioxidant activity, and microstructure of the gelatin. Gelatin obtained at 80 °C yielded 6.53% with peak gel strength (59.72 ± 4.67 g), whereas the gelatin extracted at 100 °C had the highest yield (23.24%) but the lowest gel strength (13.71 ± 3.78 g). Fourier transform infrared (FTIR) scans of gelatin derived at different temperature levels showed subtle changes in the amide region, but all the samples presented the characteristic absorption peaks of gelatin. An amino acid analysis showed high glycine (18.51%) and proline (13.45%) contents in the gelatin. Antioxidant tests revealed that gelatin obtained at 80 °C displayed the greatest scavenging effects on DPPH, hydroxyl, and ABTS radicals. Scanning electron microscopy (SEM) revealed that the gelatin made with lower temperatures showed a smoother and tighter microstructure. Rheological analyses revealed that higher extraction temperatures resulted in lower gel temperatures and melting temperatures and weaker gel network stability. The results of this study suggest that lower extraction temperatures are more suitable for the preparation of high-quality skipjack tuna bone gelatin, which gives scientific support for its application in food and medicine.

## 1. Introduction

Tuna (*Thunnus* spp.) and related species, particularly skipjack tuna (*Katsuwonus pelamis*), are highly valued in global fisheries. Skipjack tuna (*Katsuwonus pelamis*) accounts for approximately 58% of the total processed tuna catch, which exceeds 2 million metric tons per year. Tuna are rich in high-quality proteins and essential nutrients and are economically important. However, approximately 50–70% of byproducts (e.g., fish bones, black muscle, and fish skin) are generated during processing [1,2]. Tuna processing generates significant byproduct volumes, such as bones, fish skins, and offal, which are rich in high-quality protein but are often used as low-grade feed or simply thrown away. This behavior squanders valuable resources and limits corporate profitability. Researchers and industry leaders are actively exploring cutting-edge methods to transform these neglected materials into lucrative and in-demand products [3].

Collagen constitutes the primary fibrous protein in bones, cartilage, and the skin, transforming into the soluble protein gelatin through partial hydrolysis [4]. Gelatin, a versatile substance, finds diverse uses in food production, medical applications, and photographic processes. Typically, it is extracted from cow and pig skins and their bones. Since the bovine spongiform encephalopathy (BSE) outbreak, safety concerns about mammalian-derived gelatins have endured. Moreover, religious dietary restrictions and the growing popularity of vegetarianism have placed additional limitations on the use of mammalian-based gelatins. Since then, many interesting gelatin substitutes have been developed. Among numerous substitutes, fish gelatin stands out as a viable option due to its comparable characteristics to mammalian gelatin. Moreover, gelatin derived from marine animals boasts a clear edge in terms of safety due to the absence of pathogens that lead to infectious diseases. Unlike terrestrial sources, it does not harbor risks such as BSE or foot and mouth disease (FMD) [5,6]. Therefore, utilizing fish bones to produce gelatin is a viable approach. Not only does it increase the value of the byproducts generated by these processes, but it also addresses the safety concerns of gelatin and its great research interest.

The process conditions of gelatin production are a very worthy issue to explore. The process of converting collagen into gelatin is significantly influenced by temperature, a critical processing parameter. When collagen is heated under acidic or alkaline conditions, differences in temperature lead to variations in the degree of covalent cross-linking within and between collagen molecules, as well as the cleavage and hydrolysis of amide bonds. These differences can further influence the extent of peptide chains as well as the key functional attributes of gelatin, such as viscosity and gel strength [7]. By conducting in-depth studies on the properties of fish bone gelatin extracted at different temperatures, it is possible to precisely optimize the extraction process to ensure the production of gelatin products with ideal functional properties under specific temperature conditions. This approach enhances the quality and application value of fish bone gelatin, thereby better meeting the diverse needs of various industrial sectors.

In summary, in this study, Skipjack tuna bone served as the base material to study varying extraction temperature (80, 90, 100 °C) impacts on the physicochemical characteristics and gel-forming capabilities of the derived gelatin, aiming at the effective use of fish bone resources and establishing a theoretical foundation for skipjack tuna bone gelatin preparation and industrial scale production.

## 2. Materials and Methods

### 2.1. Materials and Instruments

Skipjack tuna were purchased from Hainan Yunfu Fisheries Co., Ltd. (Wenchang, China). A hydroxyl radical scavenging capacity assay kit (G0125W) was purchased from Suzhou Geruisi Biotechnology Co., Ltd. (Suzhou, China). Two different concentrations of phosphate-buffered solution (0.1 M and 0.01 M, pH 7.2–7.4) was purchased from Wuhan Procell Life Science & Technology Co., Ltd. (Wuhan, China). 2,2-Diphenyl-1-picrylhydrazyl (DPPH) and 2,2′-azino-bis (3-ethylbenzthiazoline-6-sulfonic acid) (ABTS) were obtained from Shanghai Macklin Biochemical Technology Co., Ltd. (Shanghai, China). Additional chemicals were obtained as analytical grade products from commercial vendors.

### 2.2. Preparation of Gelatin from Skipjack Tuna Bones

Vertebral samples from skipjack tuna served as the research material, were sealed in preservation bags, and then chilled to −20 °C for storage.

The specific operation method was described previously [3,8,9] with appropriate modifications. The fish vertebrae were extracted, and surface tissues were scrubbed away and rinsed with tap water to eliminate remaining flesh and contaminants. To remove noncollagen components and inhibit endogenous protease activity to protect collagen, the fish vertebrae were placed in a solution of 0.1 M NaOH in a volume ten times their weight. After 6 h, they were transferred to a 2.5% NaCl solution for 6 h, and the material was then mixed with twice its mass in distilled water, then rinsed repeatedly with distilled water and drained. To eliminate fat, the fish bones subjected to treatment were placed in a 0.1 M NaOH solution for 36 h at ambient temperature and rinsed repeatedly with pure water to pH neutral. The fish bones were combined with 0.05 M EDTA solution at a material-liquid ratio of 1:30 (*w*/*v*) in a beaker, and the decalcification time was 48 h under normal room conditions. Following decalcification, the sample was rinsed in pure water for 1 h to eliminate remaining surface acid, and then the samples were immersed in PBS at a concentration of 0.01 M (pH 7.2–7.4) for half an hour, and ultimately distilled water until pH levels stabilized at neutrality. A quantity of fish bone was placed in a conical flask and distilled water was added in a 1:10 (*w*/*v*) ratio of material to liquid. The extract was achieved through a water bath treatment at a temperature of 80, 90, or 100 °C, lasting for 13 h. At the end of extraction, samples passed through two layers of gauze, and then the extracted fluid was gathered and underwent vacuum freeze-drying.

The freeze-dried samples, labeled G80, G90, and G100 (G for gelatin; 80, 90, and 100 denote extraction temperatures), were retained as reserves at a temperature of −20 °C.

### 2.3. Determination of the Yield

The gel yield was measured following Nagarajan et al. [10], calculated as finished gelatin mass divided by skipjack tuna bone mass.

### 2.4. Determination of Moisture Content

The fish bone gelatin moisture level was measured via a rapid moisture content analyzer (HB43-S, Mettler Toledo Co., Ltd., Shanghai, China). After the instrument is zeroed, a certain amount of sample is put into the instrument for drying, and after the reading is stabilized, the value read represents the protein moisture content.

### 2.5. Gel Strength Measurement

Measurements refer to the methods of Tan et al. [11] with necessary adjustments. Initially, a certain amount of fish bone gelatin was measured out and transferred into a beaker, mixed with pure water in a certain proportion. Then, the temperature of the water bath was adjusted to 60 °C and placed the mixture in it until the gelatin was completely dissolved. This process resulted in a 6.67% gelatin solution. The solution is then refrigerated at 4 °C for approximately 16 to 18 h, during which it solidified into a gel. The gel was determined by a TA.XT Plus texture analyzer (Stable Micro Systems Co., Ltd., Godalming, UK), using a spherical probe with model number P/0.25S and the test parameters were maintained at 0.5 mm/s for speed and 4 gf for trigger force. When the probe was pressed down to 5 mm, the maximum reaction force of the gel on the probe was the gel strength (N).

### 2.6. Amino Acid Composition

Amino acid constituents were assessed using an amino acid analyzer (S-433D, Sykam Co., Ltd., Eresing, Germany). First, a 100 mg sample was sealed in an ampoule following the standard ampoule sealing procedure and the samples were hydrolyzed with 6 M HCl at 110 °C for 22 h under a nitrogen-filled environment. After hydrolysis, the concoction was strained through some filter paper. Next, the gathered fluid was carefully thinned out and then passed through a 0.22 µm filter. In the end, the precise amino acid content of the sample was obtained [12].

### 2.7. Ultraviolet (UV) Full-Wave Scanning

The UV absorption profiles of fish bone gelatin were analyzed using a multimode microplate reader (Infinite E Plex, Tecan Austria GmbH, Grödig, Austria). A 0.5 mg/mL gelatin solution in distilled water was scanned across the full wavelength spectrum from 200 to 400 nm, using distilled water as the blank reference.

### 2.8. FTIR Spectroscopy

The steps of Xue et al. [3] were followed and changed some parts with appropriate modifications. The three gelatins were scanned for infrared spectra via a Fourier transform infrared spectrometer (Nicolet 50, Thermo Nicolet Co., Ltd., Waltham, MA, USA). The samples were clamped in a FTIR spectrometer for analysis and measurement. The measurements were performed between 400 and 4000 cm^−1^. Each scan featured a 4 cm^−1^ resolution, with 32 full scans documented.

### 2.9. Measurement of Antioxidant Properties

#### 2.9.1. DPPH Radical Scavenging Capacity Analysis

Measurements refer to the methods of Wen et al. [13] with some changes. The experimental group consisted of samples mixed with an equal volume of 0.1 mM DPPH ethanol solution, the control group consisted of samples mixed with an equal volume of ethanol solution, and the blank group consisted of ethanol solution mixed with an equal volume of DPPH ethanol solution. After mixing the samples thoroughly, they were kept in the dark for 30 min. Absorbance readings at 517 nm were taken, with five repeats per test group. The sample concentration gradient was set at 2, 4, 6, 8, and 10 mg/mL. The calculation of the scavenging rate was derived using the following method:

(1) $$S\%=[1-\frac{A_{i}-A_{j}}{A_{0}}]\times 100\%$$

Here, $A_{i}$ represents the absorbance measurement obtained from a mixture of sample and DPPH, $A_{j}$ corresponds to the absorbance of sample solution combined with ethanol, and $A_{0}$ denotes the baseline absorbance, measured using ethanol mixed with DPPH solution.

#### 2.9.2. Hydroxyl Radical Scavenging Capacity Analysis

In this experiment, the ability to scavenge reactive hydroxyl radicals of the samples was quantified using a commercial hydroxyl radical detection kit [14]. In the experimental group, add 200 µL of sample, 50 µL of distilled water, and 50 µL of reagent three to the test tube. In the control group, add 200 µL of sample and 100 µL of distilled water to the test tube. In the blank group, add 250 µL of distilled water and 50 µL of reagent three to the test tube. All other operations should be strictly carried out in accordance with the provided instructions. The sample concentration gradient was set at 2, 4, 6, 8, and 10 mg/mL.

#### 2.9.3. ABTS Radical Scavenging Capacity Analysis

Measurements refer to the methods of Agrawal et al. [15] with necessary alterations. Dissolve the ABTS powder in 0.1 M PBS solution to prepare a 7 mmol/L ABTS solution. To generate ABTS free radicals, the solution was reacted with 2.45 mM potassium persulfate solution in a 1:1 volume ratio and placed in the dark for approximately 16 h without stirring. Then, the solution was diluted with the PBS solution and the absorbance at 734 nm was carefully adjusted to 0.7 ± 0.01. A 980 µL aliquot of diluted ABTS was added to the tube with 20 µL of sample and reacted for 6 min in the dark. The absorbance of the sample at 734 nm was immediately measured. The sample solution concentration levels were established at 2, 4, 6, 8, and 10 mg/mL. The scavenging rate was calculated as follows:

(2) $$S\%=[\frac{A_{C}-A_{S}}{A_{C}}]\times 100\%$$

where $A_{S}$ is the absorbance value of 20 µL of sample solution and 980 μL of ABTS solution, and $A_{C}$ is the absorbance value of 20 µL of 0.1 M PBS (pH 7.2–7.4) and 980 µL of ABTS solution.

### 2.10. Determination of Emulsification and Foaming Properties

#### 2.10.1. Emulsifying Capacity Evaluation

The measurements of emulsification properties (EAI and ESI) refer to the methods of Zamorano-Apodaca et al. [16]. The gelatin mixture was made at a 10 mg/mL ($1\times 10^{4}\;g/m^{3}$) concentration, by transferring a specific volume of soybean oil into a 50 mL centrifuge tube, then adding the gelatin solution with three times the volume of the oil. The parameters of the homogenizer (T18 Digital, IKA Co., Ltd., Staufen, Germany) were set to 15,000 r/min, and the mixture was stirred for 1 min. After stirring, take 100 µL of the emulsion that has been left to stand for 0 min and 10 min, respectively, and diluted 100 times with 0.1% SDS. After mixing thoroughly, immediately measure the absorbance at 500 nm.

The EAI is calculated via the following formula:

(3) $$\mathrm{EAI}(m^{2}/g)=\frac{2\times 2.303\times A_{0}\times N}{L\times C\times \varphi }$$

The ESI is calculated via the following formula:

(4) $$\mathrm{ESI}(\min)=\frac{A_{0}\times \Delta t}{A_{0}-A_{10}}$$

where 2 represents a constant, because the interface area of the emulsion is twice its turbidity; 2.303 represents a constant to convert absorbance into turbidity [17]; $A_{0}$ indicates the initial absorbance at 0 min; $A_{10}$ represents the absorbance measured after 10 min of emulsion; L represents optical path length of the cuvette, which is 0.01 m; N represents the dilution multiple of 100; C represents the concentration of proteins in the protein solution before the formation of the emulsion, g/$m^{3}$; φ denotes the proportion of oil within the emulsion, which is 0.25; and Δt represents 10 min.

#### 2.10.2. Foaming Capacity Evaluation

The foaming capacity (FC) and foam stability (FS) of gelatin (1 g/100 mL) were assessed following the methods of Shahidi et al. [18]. At room temperature, 10 mL ($V_{0}$) of gelatin solution was added to a 50 mL centrifuge tube and subsequently placed under a homogenizer (T18 Digital, IKA Co., Ltd., Staufen, Germany) and homogenized for 1 min at a parameter of 15,000 rpm. The total volume $V_{1}$ was then determined immediately after stirring, and after 40 min of standing, the total volume $V_{2}$ was noted.

The foaming capacity is calculated via the following formula:

(5) $$\mathrm{FC}(\%)=\frac{V_{1}-V_{0}}{V_{0}}\times 100$$

The foam stability is calculated via the following formula:

(6) $$\mathrm{FS}(\%)=\frac{V_{2}}{V_{0}}\times 100$$

### 2.11. Rheological Characteristics Measurement

Referring to the measurement method of Dileep et al. [19], a 6.67% (*w*/*v*) gelatin solution was prepared, a flat plate with a 40 mm diameter was chosen, and adjust the parameters to make the distance between the plate and the sample stage 0.5 mm. During the heating process, trimethyl silicone oil was applied to prevent water evaporation. Before conducting rheological tests, stress scanning is first performed to determine the linear viscoelastic region of the gelatin sample.

#### 2.11.1. Temperature Scan

The gel and melting points of gelatin were determined by monitoring shifts in its storage and loss modulus with temperature. And the adopted temperature control system is Peltier system. The parameters of the rheometer (Discovery HR-2, TA Instruments Co., Ltd., New Castle, DE, USA) were as follows: oscillation frequency, 1 Hz; strain, 1%. Place the sample at 40 °C for 1 min before starting the test, the scanning temperature is decreased from 40 °C to 0 °C and then increased from 0 °C up to 40 °C, the rate of temperature change during this process is 2 °C/min.

#### 2.11.2. Time Scan

The gelation rate was determined by monitoring storage and loss modulus variations over time. The rheometer settings were as follows: oscillation frequency of 1 Hz, strain of 1%, and scan time of 600 s at 4 °C. When the plate reaches 4 °C, the sample should not be left to stand, and testing should begin immediately.

### 2.12. Scanning Electron Microscopy (SEM)

Samples were examined using a field emission scanning electron microscope (Verios G4 UC, Thermo Fisher Co., Ltd., Waltham, MA, USA). We placed the gelatin solution in a vacuum freeze dryer (LGJ-12, Beijing Songyuan Huaxing Technology Development Co., Ltd., Beijing, China) and waited 48 h to obtain the freeze-dried sample. Freeze-dried samples were cut into coin-sized pieces and affixed to a tray. After gold coating, they were observed under SEM at varying magnifications [20].

### 2.13. Data Analysis

Results are shown as averages ± standard deviation, with at least three replicates for each group. Statistical evaluations were conducted in SPSS 27, and Duncan’s test (*p* < 0.05) was applied to identify significant variations. For graphical representation, all data were visualized using Origin 2024.

## 3. Experimental Outcomes and Discussion

### 3.1. Gel Yield, Moisture Content, and Gel Strength

The yield and moisture content of gelatin derived from skipjack tuna bone across various temperatures are shown in Table 1. Increased extraction temperatures led to a significant increase in gelatin output (*p* < 0.01), with a significant reduction in moisture levels (*p* < 0.01). During the extraction process, higher temperatures provide more energy to break hydrogen bonds, thus weakening the triple helix stability in collagen. This facilitates the dissolution of collagen from fish bone, resulting in increased gelatin yields [21].

Table 1 displays the varying gel strengths of gelatin derived from Skipjack tuna bones at distinct temperature extractions. Elevating the extraction temperature from 80 °C to 100 °C correlated with a statistically significant reduction in gel strength (*p* < 0.01). This aligns with the findings of Tan et al. [11] on tilapia skin gelatin gel strength at varying extraction temperatures. The gel strength is predominantly affected by its amino acid profile and the α/β chain ratio [22]. Higher extraction temperatures can degrade the α and β chains, weakening gel strength [23]. Moreover, high temperature breaks the hydrogen bonds in the gelatin molecular chains, causing the originally tight triple helix structure to unfold and the conformation to become loose. This structural change makes the gel network formed by gelatin after cooling insufficiently tight and strong, thus reducing the gel strength [24].

### 3.2. Amino Acid Determination

Table 2 presents the amino acid composition of skipjack tuna gelatin. Freeze-dried gelatin has the highest glycine content, followed by proline. Both are characteristic amino acids of gelatin. High glycine and proline levels suggest frequent repeating patterns in gelatin peptide chains [25,26]. The high levels of glycine (18.51%), proline (13.45%), and hydroxyproline (7.74%) indicate the presence of many structural proteins in skipjack tuna bone gelatin [27], which is also consistent with the typical gelatin composition [28].

The contents of glutamic acid, alanine, hydroxyproline, arginine, and aspartic acid were relatively high, whereas the contents of cystine, tyrosine, and histidine were relatively low. The amino acid profile of the skipjack tuna bone gelatin sample was generally comparable to that of gelatin derived from tuna bones [3] and that of skipjack tuna bone gelatin studied by Ding et al. [29].

### 3.3. Ultraviolet (UV) Absorption Spectra

**Figure 1:**
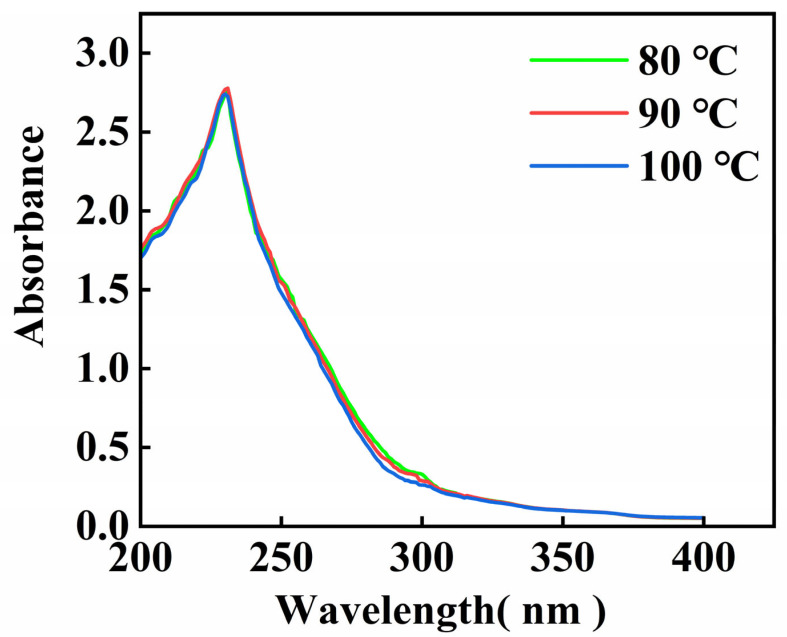
UV scanning spectrum of skipjack tuna bone gelatin.

The UV scanning results of the gelatin extracted from skipjack tuna bone are shown in Figure 1. The data matched the findings for gelatin from tilapia fish skin [30], and the maximal absorption peak of gelatin appeared at 230 nm. These absorption peaks primarily result from the n → π* transition of the C=O group of the peptide bond [31].

There is not a distinct UV absorption peak at 280 nm, suggesting that gelatin contains minimal amounts of tryptophan, tyrosine, and phenylalanine, this aligns with the findings from the amino acid analysis [32].

Gelatin UV absorption spectra changed little with extraction temperature, the UV absorption peaks of gelatin at different extraction temperatures are essentially at the same wavelengths, and only the peak sizes are slightly different. These findings indicate that the main absorption characteristics of gelatin in the ultraviolet region are relatively stable and that the extraction temperature has a limited influence on it.

### 3.4. Analysis of FTIR Spectroscopy

**Figure 2:**
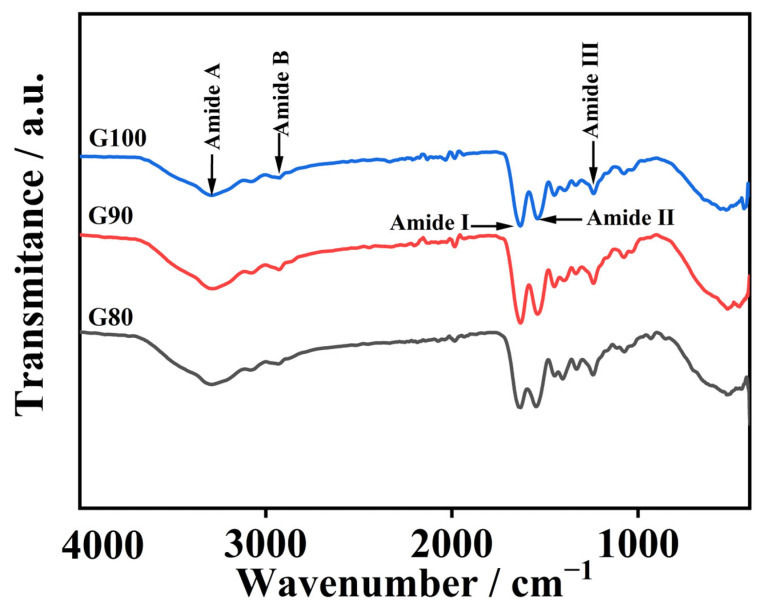
FTIR spectrum of skipjack tuna bone gelatin.

Figure 2 reveals a shift in gelatin amide I band toward higher wavenumbers with rising extraction temperatures. The amide I band position primarily reflects the C=O stretch in the peptide bond and is also slightly influenced by N-H bending vibrations, whereas the change in its specific position is closely related to hydrogen bonding and protein structural arrangement [33]. High-temperature extraction leads to the hydrolysis of gelatin molecules, producing many low-molecular-weight peptides. The C=O groups in these peptide structures become more exposed, resulting in a stronger amide I band signal [34]. Furthermore, as temperature rises, the amide I band shifts toward higher wavenumbers, which clearly indicates that triple helices break down more extensively under increasingly harsh conditions, disrupting interchain interactions [5].

The gelatin amide II band appeared between 1538 and 1546 cm^−1^. This fluctuation is associated with C-N extension of peptide bonds and bending vibrations in the N-H plane [35]. The generation of absorption peaks in the amide III band is related to the vibration of C-N and the movement of N-H in the amide bond. Furthermore, the observed phenomenon is attributed to the vibrational dynamics of the CH_2_ group within the glycine backbone and the proline side chain [10].

The gelatin amide A band appears at 3284–3293 cm^−1^ and corresponds to N-H stretching vibrations and hydrogen bonding. Usually, the amide A band usually appears between 3400 and 3440 cm^−1^, and often moves to lower wavenumbers when N-H groups in peptide bonds are involved in the formation of hydrogen bonds [36]. As temperature rose from 80 °C to 100 °C, the amide A band wavenumber dropped from 3293 to 3284 cm^−1^, and the degree of degradation of the extracted gelatin increased with increasing temperature. The more obvious the degradation occurs in gelatin, the more free amino groups are released. These free amino groups can react with other groups, causing the amide A band to shift towards a lower wavy number [37]. As the temperature increased, the amide B band changed from 2935 to 2923 cm^−1^, suggesting the occurrence of interactions between the peptide chains and the NH_3_ or CH_2_ groups [34].

Collectively, the findings show the extraction temperature markedly impacts gelatin secondary structure and chemical functionalities.

### 3.5. Analysis of Antioxidant Properties

#### 3.5.1. DPPH Radical Scavenging Capacity Test

The DPPH method assesses antioxidant capacity based on the electron-donating ability of compounds to stabilize the DPPH radical. The extent of radical neutralization is monitored by the decrease in absorbance at 517 nm, corresponding to the visible decolorization, which serves as the indicator of antioxidant capacity [38].

**Figure 3:**
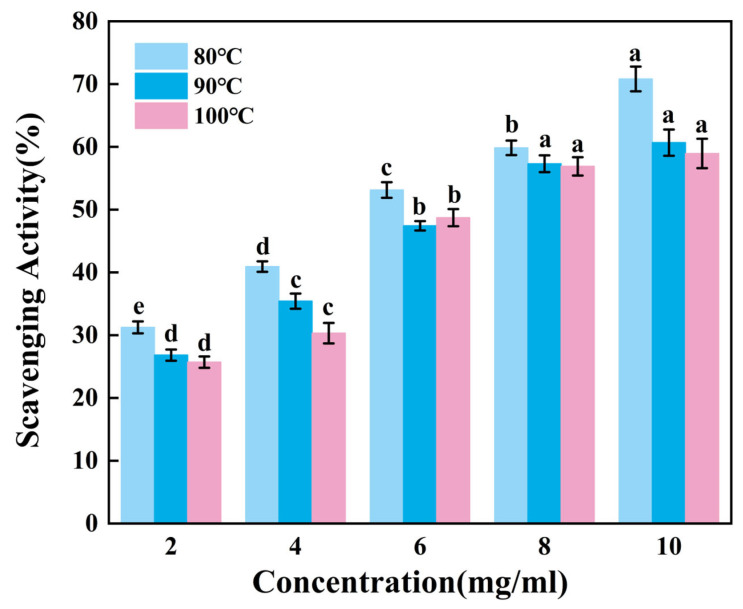
DPPH radical scavenging activity of skipjack tuna bone gelatin. Different letters above the error bar indicate significant differences between samples (*p* < 0.05).

Figure 3 presents the ability to scavenge DPPH radicals of gelatin tended to extremely significantly increase with increasing sample concentration (*p* < 0.01). Across the tested concentration range of 2–10 mg/mL, G80 consistently demonstrated superior scavenging activity compared to others. Within the concentration range of 8–10 mg/mL, there was no significant change in the scavenging capacity of G90 and G100. While G90 and G100 showed comparable performance throughout most of this range, G90 maintained a marginal advantage over G100 in terms of scavenging efficiency. Therefore, the DPPH radical scavenging ability of skipjack tuna bone gelatin may decrease with increasing extraction temperature in higher temperature ranges.

Gelatin extracted at lower temperatures exhibited higher DPPH radical scavenging activity, suggesting that gelatin may contain bioactive components. These components may exert antioxidant effects in biological systems, helping to protect cells from oxidative stress damage [39].

#### 3.5.2. Hydroxyl Radical Scavenging Capacity Test

Hydroxyl radicals are the most reactive radicals. They form from superoxide anion (O_2_^−^) and hydrogen peroxide (H_2_O_2_). This reaction requires metal ions like copper or iron. Hydroxyl radicals cause severe damage in free radical pathology. They attack nearly all molecules within living cells. Thus, it is necessary to study the ability of gelatin to scavenge Hydroxyl radicals [40].

**Figure 4:**
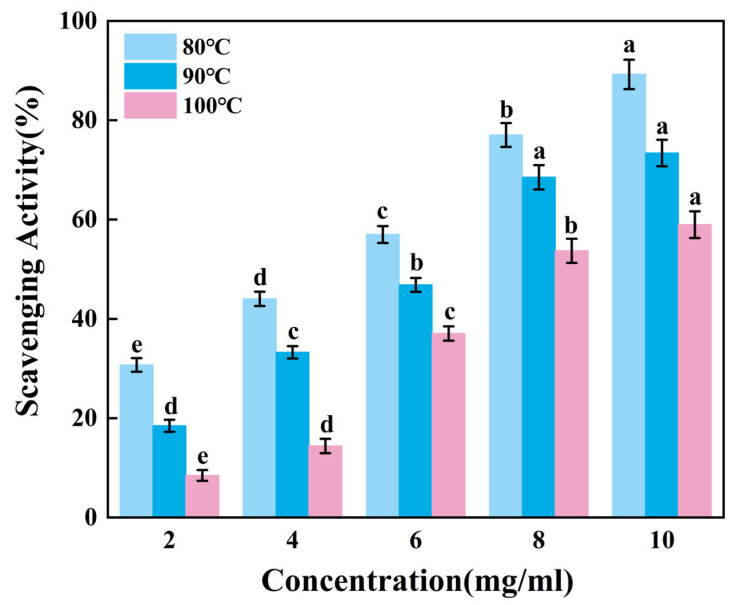
Hydroxyl radical scavenging activity of skipjack tuna bone gelatin. Different letters above the error bar indicate significant differences between samples (*p* < 0.05).

Figure 4 revealed that the ability to scavenge reactive hydroxyl radicals of gelatin tended to increase extremely significantly with higher sample mixture concentrations (*p* < 0.01). When the concentration increases from 8 mg/mL to 10 mg/mL, the scavenging rates of G80 and G100 change significantly, while the scavenging rate of G90 shows no obvious increase. Apparently, skipjack tuna bone gelatin has a strong ability to scavenge hydroxyl radicals, with the highest scavenging rate of G80 and the lowest scavenging rate of G100. At elevated temperatures, increased extraction heat may reduce the ability of gelatin to scavenge hydroxyl radicals.

Therefore, gelatin extracted at lower temperatures can more effectively reduce hydroxyl radical concentrations, thereby inhibiting oxidative damage caused by free radicals.

#### 3.5.3. ABTS Radical Scavenging Capacity Test

ABTS radicals exhibit a distinct absorption peak at a wavelength of 734 nm, and their solution appears blue-green in color. When antioxidants react with ABTS radicals, the radicals are reduced, causing the solution color to lighten and the absorbance to decrease accordingly. By measuring the change in absorbance at 734 nm before and after the reaction, the activity of the antioxidant can be quantitatively analyzed [41].

**Figure 5:**
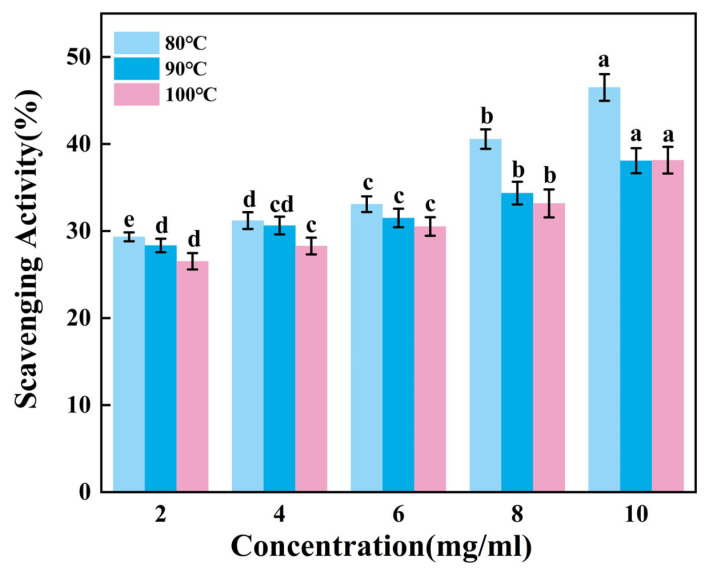
ABTS radical scavenging activity of skipjack tuna bone gelatin. Different letters above the error bar indicate significant differences between samples (*p* < 0.05).

According to Figure 5, the ability of gelatin to scavenge ABTS radicals increases extremely significantly as the sample concentration rose (*p* < 0.01). Within the sample concentration range of 2–6 mg/mL, the scavenging efficiency of G90 shows no significant change (*p* > 0.05). As the concentration increases from 8 mg/mL to 10 mg/mL, the scavenging efficiency of G80 significantly improves, while the scavenging efficiencies of G90 and G100 show no significant changes. It can be inferred that gelatin has a relatively weak ability to scavenge ABTS free radicals, and the improvement in scavenging ability is limited when the concentration increases. Additionally, this ability may further weaken as the extraction temperature increases.

The ABTS radical scavenging ability of gelatin is relatively poor compared to hydroxyl radicals and DPPH radicals. This may be due to the fact that the active components in gelatin have a weaker reaction ability with ABTS radicals. This difference may be the result of a combination of factors, including the chemical structure, composition, source, purity, modification, and experimental conditions of gelatin.

### 3.6. Foaming and Emulsifying Properties

According to Table 3, both the foaming ability and foam stability of the gelatin samples saw a notable rise (*p* < 0.01) as the derived temperature went up. The way molecules move, penetrate, and reorganize at the air–water boundary plays a key role in shaping their foaming characteristics [42]. The proteins in the dispersion, when they are at the air-liquid interface, reduce the interfacial tension, which in turn generates foam. A higher extraction temperature may enable protein molecules to enrich the air-liquid interface more quickly during foaming, which is conducive to lowering the surface tension and promoting the adsorption of gelatin at the air-liquid interface, thus improving the foaming performance. Additionally, enhanced interfacial adsorption could drive gelatin particle aggregation, expansion, and structural rearrangement at the interface, resulting in an increase in the interaction between interfacial gelatins and thus improving foaming stability [43,44].

Table 3 reveals a decrease in EAI (*p* < 0.05) and a significant decline in ESI (*p* < 0.01) for gelatin as extraction temperatures rise. Higher extraction temperatures caused gelatin emulsions to collide more often. Furthermore, flocculation and coalescence levels in the gelatin emulsion increase, which results in a decrease in the emulsification properties of the gelatin emulsion [45]. Therefore, G80 showed better emulsifying activity and emulsion stability than did G100.

Table 3 shows that the gelatin solution has strong emulsification stability at different extraction temperatures. Gelatin molecules that are rich in hydrophilic and hydrophobic amino acids can form a stable adsorption layer at the oil-water interface. This adsorption layer can effectively reduce the interfacial tension and prevent the aggregation and merging of droplets through electrostatic repulsion, thus enhancing the emulsion stability [46]. An amino acid analysis revealed that gelatin contains high contents of glycine, alanine, glutamic acid, and aspartic acid, which is one of the reasons for the strong emulsion stability of the gelatin solution.

Gelatin is a polymeric material with charged and amphiphilic groups, thus exhibiting outstanding surface activity, particularly in emulsification and foaming. Gelatin’s foaming ability is quite strong, primarily because it effectively reduces the surface tension at the liquid–gas interface. Additionally, gelatin can form a three-dimensional network structure, enhancing the stability of the continuous phase, thereby aiding in foam stability. Therefore, in food processing, the role of gelatin cannot be underestimated, as it can be used in the production of candies and to enhance the texture and structure of products such as bread [47].

### 3.7. Rheological Characterization Analysis

#### 3.7.1. Temperature Scan

**Figure 6:**
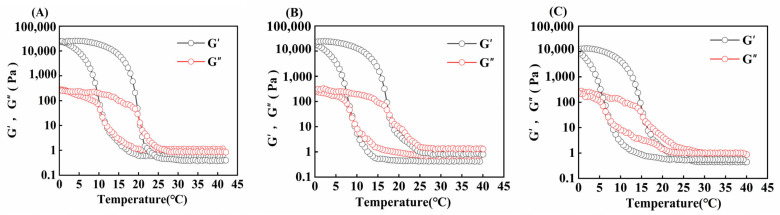
Gel and melting temperature of skipjack tuna bone gelatin at extraction temperatures of 80 °C (**A**), 90 °C (**B**), and 100 °C (**C**).

The variation in G′ (energy storage modulus) and G″ (loss modulus) of gelatin obtained from different extraction temperatures is shown in Figure 6. When the modulus curves intersect during the cooling process, it represents the temperature (T_g_) at which the gelatin transforms from the liquid state to the gel state, whereas when they intersect during the heating process, it represents the temperature (T_m_) at which the gelatin transforms from the gel state to the liquid state [48].

In Table 4, the gel and melting points of skipjack tuna bone gelatin declined progressively as extraction temperature rose (*p* < 0.05). When the derived temperature elevated, the degree of gelatin structure disruption elevated, and the number of high-molecular-weight subunits gradually decreased, whereas the number of low-molecular-weight fragments increased, which made it difficult to construct a stable gel network and thus weakened its stability during heat treatment [11].

Moreover, varying extraction temperatures can influence the concentration of amino acids in gelatin. A higher amino acid content enhances the stability of the gelatin’s triple-helix structure, which in turn improves its rheological performance. As a result, such gelatin tends to have superior gel strength as well as higher melting and gel temperatures [49].

#### 3.7.2. Time Scan

**Figure 7:**
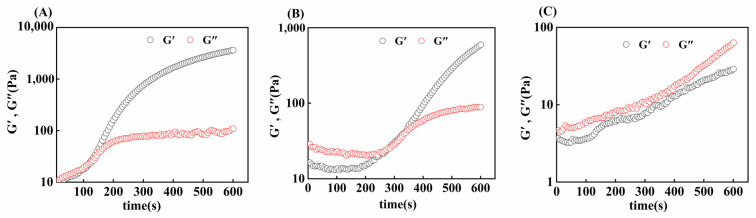
Gel time of skipjack tuna bone gelatin at extraction temperatures of 80 °C (**A**), 90 °C (**B**), and 100 °C (**C**).

The gelation rates of gelatin obtained from three different extraction temperatures were compared via time scanning. As shown in Figure 7, initially, the G′ of the gel sample was smaller than the G″, and with increasing time, the G′ of the gel sample increased rapidly, and then, the intersection of G′ and G″ appeared, which was the time required for the gel formation of skipjack tuna bone gelatin at 4 °C. At this time, the sample changed from a liquid form to a solid-like form, a denser and more elastic network formed inside the gelatin, and the elastic properties of the gelatin increased [50].

G80 took less time to form a gel (121 ± 7 s) than G90 did (273 ± 9 s), and G100 takes longer to form a gel or cannot form a gel. The gelatin extracted at higher temperatures was more susceptible to thermal degradation, leading to a greater degree of degradation. This degradation makes the alignment and joining of gelatin molecular chains more difficult, thus prolonging the formation of the gel network [35]. Thus, we can conclude that different extraction temperatures affect the gelation time of skipjack tuna bone gelatin.

### 3.8. Microstructure of the Gelatin

**Figure 8:**
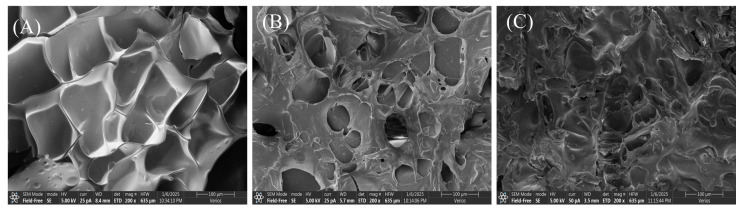
Microstructure of skipjack tuna bone gelatin extracted at 80 °C (**A**), 90 °C (**B**), and 100 °C (**C**).

Figure 8 shows the microstructure of gelatin at different extraction temperatures. Skipjack tuna bone gelatin has a loose structure with more pores and uneven surfaces, which may be due to dehydration during freeze-drying [51]. G80 was arranged in an orderly fashion, and the pore sizes were essentially the same, whereas G90 and G100 were arranged in an unorganized fashion, and the pore sizes varied. This resembles the microstructure observed in perch skin gelatin, with larger cavities appearing in samples extracted at elevated temperatures, while gelatin obtained at cooler temperatures exhibited a more uniformly fine and orderly pattern [35]. Differences in pore structure may affect the physical properties of gelatin, such as water absorption and mechanical strength. Extraction conditions have a significant impact on gelatin structure. Gelatin extracted under milder conditions retains more intact chains, and these polypeptide chains form a dense three-dimensional network structure through stronger covalent bonds, giving gelatin with a more ordered microstructure [36]. The distribution of molecular weight in gelatin also impacts its microscopic structure. The higher the content of high-molecular-weight peptides in gelatin is, the tighter the gel structure and the finer the internal structure [52]. This suggests that molecular weight distribution not only affects the microstructure of gelatin but may also influence its functional properties.

In the food industry, gelatin performance is crucial for stability and texture in food products. Gelatin with fewer pores and an ordered pore arrangement may exhibit superior gel properties, making it suitable for applications such as jellies and puddings.

## 4. Conclusions

This study revealed that the extraction temperature strongly affects the quality of the gelatin extracted from skipjack tuna bones. The gelatin obtained at 80 °C exhibited the peak gel strength and maximum antioxidant capacity because it retained more of its natural structure. Higher temperatures increased the yield but broke down the gelatin structure, leading to weak gels and lower heat resistance. Thus, using 80 °C is best for making high-quality gelatin from tuna bones. This method can be used in the food industry, health products, and biomedical materials while reducing fish processing waste and supporting eco-friendly practices.

## Figures and Tables

**Table 1:** Gel strength, yield, and moisture content of skipjack tuna bone gelatin.

	Gel Strength (N)	Yield (%)	Moisture Content (%)
100 °C	0.13 ± 0.04 ^c^	23.2 ± 0.6 ^a^	12.0 ± 0.3 ^c^
90 °C	0.31 ± 0.02 ^b^	12.4 ± 0.7 ^b^	13.0 ± 0.4 ^b^
80 °C	0.59 ± 0.05 ^a^	6.5 ± 0.5 ^c^	15.2 ± 0.2 ^a^

Distinct lowercase letters in a column denote significant variations (*p* < 0.05).

**Table 2:** Amino acid composition of skipjack tuna bone gelatin.

Number	Amino Acid	Content
1	Asp	5.56
2	Glu	9.96
3	Ser	2.84
4	His	0.66
5	Gly	18.51
6	Thr	2.25
7	Ala	8.77
8	Arg	7.27
9	Tyr	0.53
10	Cys	0.01
11	Val	2.31
12	Met	1.57
13	Phe	1.83
14	Ile	1.22
15	Leu	2.55
16	Lys	2.57
17	Pro	13.45
18	Hyp	7.74
19	Total	89.60

**Table 3:** Foaming and emulsifying properties of skipjack tuna bone gelatin.

	Foaming Capacity (%)	Foaming Stability (%)	Emulsifying Activity (m^2^/g)	Emulsion Stability (min)
80 °C	25 ± 2 ^c^	101 ± 5 ^b^	36 ± 2 ^a^	121 ± 2 ^a^
90 °C	53 ± 4 ^b^	126 ± 5 ^a^	30 ± 2 ^b^	115 ± 2 ^b^
100 °C	75 ± 5 ^a^	134 ± 7 ^a^	28 ± 2 ^b^	106 ± 3 ^c^

Distinct lowercase letters in a column denote significant variations (*p* < 0.05).

**Table 4:** Gelling temp. and melting temp. of skipjack tuna bone gelatin.

	Gelling Temperature (°C)	Melting Temperature (°C)
80 °C	12 ± 1 ^a^	20 ± 1 ^a^
90 °C	9 ± 1 ^b^	18 ± 1 ^b^
100 °C	7 ± 1 ^b^	16 ± 1 ^b^

Distinct lowercase letters in a column denote significant variations (*p* < 0.05).

## Data Availability

The original contributions presented in this study are included in the article. Further inquiries can be directed to the corresponding author.

## References

[1] Xiang X.W., Zhou X.L., Wang R., Shu C.H., Zhou Y.F., Ying X.G., Zheng B. (2021). Protective Effect of Tuna Bioactive Peptide on Dextran Sulfate Sodium-Induced Colitis in Mice. Mar. Drugs.

[2] Pinrattananon S., Courtes F., Chorhirankul N., Payongsri P., Pongtharangkul T., Janssen A.E.M., Niamsiri N. (2023). The Effect of Different pH Conditions on Peptides’ Separation from the Skipjack Dark Meat Hydrolysate Using Ceramic Ultrafiltration. Foods.

[3] Xue J., Xu F., Lu W., Yang L., Liang J., Mao P., Chen L., Yang H., Chen K., Wang Z. (2025). Development and characterization of gelatin peptides and peptide-calcium chelates from tuna processing byproducts of skins and bones. Food Chem..

[4] Gómez-Guillén M.C., Giménez B., López-Caballero M.E., Montero M.P. (2011). Functional and bioactive properties of collagen and gelatin from alternative sources: A review. Food Hydrocoll..

[5] Kaewruang P., Benjakul S., Prodpran T., Nalinanon S. (2013). Physicochemical and functional properties of gelatin from the skin of unicorn leatherjacket (*Aluterus monoceros*) as affected by extraction conditions. Food Biosci..

[6] Sha X.-M., Tu Z.-C., Liu W., Wang H., Shi Y., Huang T., Man Z.-Z. (2014). Effect of ammonium sulfate fractional precipitation on gel strength and characteristics of gelatin from bighead carp (*Hypophthalmichthys nobilis*) scale. Food Hydrocoll..

[7] Karim A.A., Bhat R. (2009). Fish gelatin: Properties, challenges, and prospects as an alternative to mammalian gelatins. Food Hydrocoll..

[8] Nagai T., Suzuki N. (2000). Isolation of collagen from fish waste material—Skin, bone and fins. Food Chem..

[9] Yang X.R., Zhao Y.Q., Qiu Y.T., Chi C.F., Wang B. (2019). Preparation and Characterization of Gelatin and Antioxidant Peptides from Gelatin Hydrolysate of Skipjack Tuna (*Katsuwonus pelamis*) Bone Stimulated by in vitro Gastrointestinal Digestion. Mar. Drugs.

[10] Nagarajan M., Benjakul S., Prodpran T., Songtipya P., Kishimura H. (2012). Characteristics and functional properties of gelatin from splendid squid (*Loligo formosana*) skin as affected by extraction temperatures. Food Hydrocoll..

[11] Tan C.C., Karim A.A., Uthumporn U., Ghazali F.C. (2019). Effect of Extraction Temperature on the Physicochemical Properties of Gelatin from the Skin of Black Tilapia (*Oreochromis mossambicus*). J. Phys. Sci..

[12] Hu S., Yuan J., Gao J., Wu Y., Chen H. (2020). Antioxidant and anti-inflammatory potential of peptides derived from the in vitro gastrointestinal digestion of germinated and heat-treated foxtail millet (*Setaria italica*) proteins. J. Agric. Food Chem..

[13] Wen C., Zhang J., Zhang H., Duan Y., Ma H. (2019). Effects of divergent ultrasound pretreatment on the structure of watermelon seed protein and the antioxidant activity of its hydrolysates. Food Chem..

[14] Suzhou Gerusici Bio-Technology Co., Ltd. (2023). Hydroxyl Radical Scavenging Capacity Assay Kit (Cat. No. G0125W) [Assay Kit]. https://www.geruisi-bio.com.

[15] Agrawal H., Joshi R., Gupta M. (2016). Isolation, purification and characterization of antioxidative peptide of pearl millet (Pennisetum glaucum) protein hydrolysate. Food Chem..

[16] Zamorano-Apodaca J.C., García-Sifuentes C.O., Carvajal-Millán E., Vallejo-Galland B., Scheuren-Acevedo S.M., Lugo-Sánchez M.E. (2020). Biological and functional properties of peptide fractions obtained from collagen hydrolysate derived from mixed byproducts of different fish species. Food Chem..

[17] Pearce K.N., Kinsella J.E. (1978). Emulsifying properties of proteins: Evaluation of a turbidimetric technique. J. Agric. Food Chem..

[18] Shahidi F., Han X.Q., Synowiecki J. (1995). Production and characteristics of protein hydrolysates from capelin (*Mallotus villosus*). Food Chem..

[19] Dileep A., Shamasundar B., Binsi P., Badii F., Howell N. (2006). Effect of Ice Storage on the Physicochemical and Dynamic Viscoelastic Properties of Ribbonfish (*Trichiurus* spp.) Meat. J. Food Sci..

[20] Pei W., Hua Z., Zhenyu S., Dan L.I., Yanling X. (2019). Isolation and Characterization of Acid-soluble Collagen and Pepsin-soluble Collagen from the Skin of Hybrid Sturgeon. J. Wuhan Univ. Technol.-Mater. Sci. Ed..

[21] Sinthusamran S., Benjakul S., Hemar Y., Kishimura H. (2016). Characteristics and Properties of Gelatin from Seabass (*Lates calcarifer*) Swim Bladder: Impact of Extraction Temperatures. Waste Biomass Valorization.

[22] Moosavi-Nasab M., Yazdnai-Dehnavi M., Mirzapour-Kouhdasht A. (2020). The effects of enzymatically aided acid swelling process on gelatin extracted from fish by-products. Food Sci. Nutr..

[23] Sha X.M., Hu Z.Z., Ye Y.H., Xu H., Tu Z.C. (2019). Effect of extraction temperature on the gelling properties and identification of porcine gelatin. Food Hydrocoll..

[24] Wang Y., Zhang L., Cao G., Li Z., Du M. (2024). Effect of Heat Treatment on Gelatin Properties and the Construction of High Internal Phase Emulsions for 3D Printing. Foods.

[25] Blidi O.E., Omari N.E., Balahbib A., Ghchime R., Barkiyou M. (2021). Extraction Methods, Characterization and Biomedical Applications of Collagen: A Review. Biointerface Res. Appl. Chem..

[26] Lin Y., Cai X., Wu X., Lin S., Wang S. (2019). Fabrication of snapper fish scales protein hydrolysate-calcium complex and the promotion in calcium cellular uptake. J. Funct. Foods.

[27] Makgobole M.U., Onwubu S.C., Baruwa A., Mpofana N., Obiechefu Z., Naidoo D., Khathi A., Mkhwanazi B. (2024). Optimization of Collagen Extraction from Fish Scales Using Tris-Glycine Buffer: A Taguchi Methodological Approach. Mar. Drugs.

[28] Bae I., Osatomi K., Yoshida A., Osako K., Yamaguchi A., Hara K. (2008). Biochemical properties of acid-soluble collagens extracted from the skins of underutilized fishes. Food Chem..

[29] Ding D., Du B., Zhang C., Fakhar Z., Yaqin H. (2019). Isolation and identification of an antioxidant collagen peptide from skipjack tuna (*Katsuwonus pelamis*) bone. RSC Adv..

[30] Song Z., Liu H., Chen L., Chen L., Zhou C., Hong P., Deng C. (2020). Characterization and comparison of collagen extracted from the skin of the Nile tilapia by fermentation and chemical pretreatment—ScienceDirect. Food Chem..

[31] Yan M., Li B., Zhao X., Ren G., Zhuang Y., Hou H., Zhang X., Chen L., Fan Y. (2008). Characterization of acid-soluble collagen from the skin of walleye pollock (*Theragra chalcogramma*). Food Chem..

[32] Yang Y.N., Li C.Y., Song W., Wang W., Qian G. (2016). Purification, optimization and physicochemical properties of collagen from soft-shelled turtle calipash. Int. J. Biol. Macromol..

[33] Sarbon N.M., Badii F., Howell N.K. (2013). Preparation and characterization of chicken skin gelatin as an alternative to mammalian gelatin. Food Hydrocoll..

[34] Liu Y., Xia L., Jia H., Li Q., Jin W., Dong X., Pan J. (2017). Physiochemical and functional properties of chum salmon (*Oncorhynchus keta*) skin gelatin extracted at different temperatures. J. Sci. Food Agric..

[35] Sinthusamran S., Benjakul S., Kishimura H. (2014). Characteristics and gel properties of gelatin from skin of seabass (*Lates calcarifer*) as influenced by extraction conditions. Food Chem..

[36] Kittiphattanabawon P., Benjakul S., Sinthusamran S., Kishimura H. (2016). Gelatin from clown featherback skin: Extraction conditions. LWT-Food Sci. Technol..

[37] Pan J., Li Q., Jia H., Xia L., Jin W.G., Shang M.J., Xu C., Dong X.P. (2017). Physiochemical and functional properties of tiger puffer (*Takifugu rubripes*) skin gelatin as affected by extraction conditions. Int. J. Biol. Macromol..

[38] Kanwate B.W., Patel K., Karkal S.S., Rajoriya D., Sharan K., Kudre T.G. (2024). Production of Antioxidant, Angiotensin-Converting Enzyme Inhibitory and Osteogenic Gelatin Hydrolysate from Labeo rohita Swim Bladder. Mar. Biotechnol..

[39] Baliyan S., Mukherjee R., Priyadarshini A., Vibhuti A., Gupta A., Pandey R.P., Chang C.M. (2022). Determination of Antioxidants by DPPH Radical Scavenging Activity and Quantitative Phytochemical Analysis of Ficus religiosa. Molecules.

[40] Shen Q., Ou A., Liu S., Elango J., Wang S., Henriques T., Wu W., Robinson J., Bao B. (2019). Effects of ion concentrations on the hydroxyl radical scavenging rate and reducing power of fish collagen peptides. J. Food Biochem..

[41] Cano A., Maestre A.B., Hernández-Ruiz J., Arnao M.B. (2023). ABTS/TAC Methodology: Main Milestones and Recent Applications. Processes.

[42] Elavarasan K., Kumar V.N., Shamasundar B.A. (2013). Antioxidant and Functional Properties of Fish Protein Hydrolysates from Fresh Water Carp (*Catla catla*) as Influenced by the Nature of Enzyme. J. Food Process. Preserv..

[43] Tanuja S., Viji P., Zynudheen A., Arnao M.B. (2012). Composition, functional properties and antioxidative activity of hydrolysates prepared from the frame meat of Striped catfish (*Pangasianodon hypophthalmus*). Egypt. J. Biol..

[44] Feng X., Zhu H., Yu Y., Dai H., Ma L., Zhang Y. (2025). Effect of microwave extraction temperatures on the gelling, foaming and emulsifying characteristics of Bigeye Tuna (*Thunnus obesus*) fish skin gelatin. Food Biosci..

[45] Tan C.C., Karim A.A., Uthumporn U., Ghazali F.C. (2020). Effect extraction temperature on the emulsifying properties of gelatin from black tilapia (*Oreochromis mossambicus*) skin. Food Hydrocoll..

[46] Farooq S., Ahmad M.I., Zheng S., Ali U., Li Y., Shixiu C., Zhang H. (2024). A review on marine collagen: Sources, extraction methods, colloids properties, and food applications. Collagen Leather.

[47] Ahmad M.I., Li Y., Pan J., Liu F., Dai H., Fu Y., Huang T., Farooq S., Zhang H. (2024). Collagen and gelatin: Structure, properties, and applications in food industry. Int. J. Biol. Macromol..

[48] Anvari M., Chung D. (2016). Dynamic rheological and structural characterization of fish gelatin-Gum arabic coacervate gels cross-linked by tannic acid. Food Hydrocoll..

[49] Aykin-Dincer E., Koc A., Erbas M. (2017). Extraction and physicochemical characterization of broiler (*Gallus gallus domesticus*) skin gelatin compared to commercial bovine gelatin. Poult. Sci..

[50] Zhu B., Xin C., Li J., Li B. (2019). Ultrasonic Degradation of Konjac Glucomannan and the Effect of Freezing Combined with Alkali Treatment on Their Rheological Profiles. Molecules.

[51] Wang L., Liang Q., Chen T., Wang Z., Xu J., Ma H. (2014). Characterization of collagen from the skin of Amur sturgeon (*Acipenser schrenckii*). Food Hydrocoll..

[52] Zhang J., Duan R., Wang Y., Yan B., Xue W. (2012). Seasonal differences in the properties of gelatins extracted from skin of silver carp (*Hypophthalmichthys molitrix*). Food Hydrocoll..

